# A general class of improved population variance estimators under non-sampling errors using calibrated weights in stratified sampling

**DOI:** 10.1038/s41598-023-47234-1

**Published:** 2024-02-05

**Authors:** M. K. Pandey, G. N. Singh, Tolga Zaman, Aned Al Mutairi, Manahil SidAhmed Mustafa

**Affiliations:** 1 Department of Mathematics and Computing, Indian Institute of Technology (Indian School of Mines), Dhanbad, 826004 India; 2 Faculty of Health Sciences, Gumushane University, Gumushane, Turkey; 3 Department of Mathematical Sciences, College of Science, Princess Nourah bint Abdulrahman University, P.O. Box 84428, Riyadh, 11671 Saudi Arabia; 4 Department of Statistics, Faculty of Science, University of Tabuk, Tabuk, Saudi Arabia

**Keywords:** Engineering, Mathematics and computing

## Abstract

This paper proposes a new calibration estimator for population variance within a stratified two-phase sampling design. It takes into account random non-response and measurement errors, specifically applying this method to estimate the variance in Gas turbine exhaust pressure data. The study integrates additional information from two highly positively correlated auxiliary variables to develop a general class of estimators tailored for the stratified two-phase sampling scheme. The properties of these estimators, in terms of their biases and mean square errors, have been thoroughly examined and extensively analyzed through numerical and simulation studies. Furthermore, the calibrated weights of the strata are derived. The proposed estimators outperform the natural estimator of population variance. Finally, suitable recommendations have been made for survey statisticians intending to apply these findings to real-life problems.

## Introduction

In many practical scenarios, estimating population variance is a crucial task with wide-ranging applications, spanning various domains including finance, healthcare, and weather forecasting. Actuaries and insurance analysts heavily rely on population variance estimation to make well-informed decisions. In the realm of weather forecasting, grasping the variability in temperature, humidity, and other meteorological factors at diverse locations is fundamental for precise predictions. To bolster the precision of estimators in sample surveys, auxiliary variables play a pivotal role. For instance, when estimating crop yields, incorporating data on the area covered by crops can significantly enhance prediction accuracy. Numerous studies, such as^1^ did work on the use of auxiliary information in estimating the finite population variance^2^, developed a class of estimators using auxiliary information for estimating finite population variance, and^3^ introduced a new procedure for variance estimation in simple random sampling using auxiliary information^4^. further improved the estimation of finite population variance using dual supplementary information under stratified random sampling, while^6^ explored the more efficient use of auxiliary information in population variance estimation, presenting a new family of estimators.

Moreover, recent research has delved into variance estimation using auxiliary information, with innovative approaches like memory type ratio and product estimators^7,8^ gaining attention. These endeavors aim to enhance the accuracy and reliability of population variance estimation in diverse sampling designs.

However, sample surveys often encounter practical challenges that result in non-response or missing data. These challenges encompass non-contact, refusal to cooperate, and various other reasons. When a substantial amount of data goes missing, it casts doubt on the reliability of ensuing statistical results. Diverse types of missing data patterns, such as missing at random (MAR) and missing completely at random (MCAR), can be observed. Particularly noteworthy is the MAR pattern, characterized by the probability of missingness being independent of the unobserved data’s value.

In the presence of random non-response or measurement errors, various researchers have addressed the need for robust estimators^9^. introduced a class of estimators using auxiliary information for estimating finite population variance in the presence of measurement errors, while^10^ developed classes of factor-type estimators in the presence of measurement error^11^. focused on the estimation of the population coefficient of variation in the presence of measurement errors, and^12^ worked on estimating the population mean in the presence of measurement error and non-response under stratified random sampling^13^. contributed to the estimation of the finite population distribution function with the dual use of auxiliary information under non-response, and^14^ introduced a generalized class of estimators for sensitive variables in the presence of measurement error and non-response^15^. explored the estimation of finite population mean using dual auxiliary variables for non-response using simple random sampling, while^16^ and Bhushan (2023) proposed classes of robust estimators to handle correlated measurement errors and new logarithmic type imputation techniques in presence of measurement errors within the survey sampling literature. These errors may stem from flawed measuring instruments, shortcomings in survey methodology, vague questionnaires, or imprecise measurements.

The calibration approach, pioneered by^18^, has garnered prominence in statistical practice. Its objective is to devise unbiased estimation procedures with minimal dispersion, leveraging auxiliary variables. Subsequent researchers, exemplified by^19^ and^20^, have fine-tuned and extended calibration estimation procedures, striving to minimize the divergence between initial and final weights while adhering to calibration equations and constraints.

Recent advances in calibration techniques, as demonstrated by^21^, have focused on a class of calibration estimators under stratified random sampling in the presence of various kinds of non-sampling errors^22^. Explored calibration estimation for ratio estimators in stratified sampling for proportion allocation, and^23^ further advanced the finite population distribution function estimation with the dual use of auxiliary information under simple and stratified random sampling^5^. investigated the use of dual ancillary variables to estimate the population mean under stratified random sampling, while^24^ worked on modified estimators of the finite population distribution function based on the dual use of auxiliary information under stratified random sampling. These techniques have streamlined the optimization of stratum weights in stratified random sampling, ultimately refining estimates, particularly when closely related auxiliary variables are integrated.

To underscore the practical significance of this research, let’s consider real-life examples:

1. In healthcare research, when conducting patient surveys to evaluate the effectiveness of medical treatments, not all patients may respond, and measurement errors can occur due to self-reporting. Accurate population variance estimation in such cases is crucial to making informed decisions about treatment strategies.
2. In market research, understanding consumer preferences through surveys is essential for product development and marketing strategies. Non-response from certain demographic groups or errors in survey responses can distort the estimation of market variances, impacting business decisions.
3. In educational assessments, when evaluating the performance of schools or educational programs, student participation may vary, and measurement errors can affect the assessment outcomes. Reliable population variance estimation is vital for making informed policy decisions and improving education quality.
4. By addressing these issues across diverse fields, this innovative framework aims to provide a reliable approach for accurately estimating population variances, thereby enhancing decision-making processes in real-life scenarios. Additionally, the proposed estimation strategy may be applied to estimate the variance in Gas turbine exhaust pressure, as illustrated using real data in a subsequent section of the manuscript.

Survey sampling necessitates addressing uncertainty and imprecision. Neutrosophic statistics, championed by^25^ in ’Neutrosophy: Neutrosophic Probability, Set, and Logic: Analytic Synthesis & Synthetic Analysis,’ extend classical statistics for indeterminate data. Aslam’s contributions include ’A New Sampling Plan using Neutrosophic Process Loss Consideration’^26^ and ’Neutrosophic Analysis of Variance: Application to University Students’^27^, among others, illustrating its application in handling vague and imprecise observations in populations or samples.

Motivated by the aforementioned discussions, the present work proposes a wide class of estimators of population variance in two-phase sampling for the stratified population in the presence of random non-response and measurement errors in sample data. The stratum weights have been optimized using calibration procedures, which enables us to get more accurate estimates of the population variance. The performances of the suggested class of estimators have been deeply examined through empirical and simulation studies.

## Sample structure

Consider a finite population of size N divided into L non-overlapping strata, each containing $N_k$(k=1,2,..., L) units. Let Y, X, and Z be the study variable, first and second auxiliary variables, respectively. Let $y_{ki}$, $x_{ki}$, and $z_{ki}$ be the ith values of y, x, and z for the k-th (k = 1, 2,..., L) stratum. To estimate the population variance of the study variable Y, It is assumed that the information on the second auxiliary Z is readily available for all the population units. Hence its population variance is known. However, information on the first auxiliary variable is not available for all the units of the population. It is also assumed that the random non-response is observed in the sample data on the study and first auxiliary variables Y and X, respectively. In the first phase, a sample, say $S_{{n}_k}$ of size $n_k$ (k=1,2..., L), is drawn from kth strata using simple random sampling without replacement and observed for the variables y and x. Let in the first phase sample of size $n_k$, $n_{k-r_{1k}}$ respond and random non-response observed on the $r_{1k}$ units. Again in the second phase, from the $n_{k-r_{1k}}$ respondent units, another simple random sample without replacement, say $S_{m_k}$, of size $m_k$, is chosen from which $m_{k-r_{2k}}$ units respond and $r_{2k}$ units do not respond.

### Notations

From now on, we will use the following notations:

$\sigma ^2_Y$: The population variance of Y, i.e, the characteristics under study

$S^2_{Y_{N_k}}$=$\frac{1}{N_k-1}\sum _{j=1}^{N_k}(Y_{kj}-\bar{Y}_{N_k})^2$: The population mean squares of the kth stratum of the study variable Y.

$S^2_{X_{N_k}}$=$\frac{1}{N_k-1}\sum _{j=1}^{N_k}(X_{kj}-\bar{X}_{N_k})^2$, $S^2_{Z_{N_k}}$=$\frac{1}{N_k-1}\sum _{j=1}^{N_k}(Z_{kj}-\bar{Z}_{N_k})^2$: The population mean squares of the kth stratum for the auxiliary variables X and Z, respectively.

$s^{*^2}_{x_{n_k}}=\frac{1}{n_k-r_{1k}-1}\sum ^{n_k-r_{1k}}_{j=1}(x_{kj}-\bar{x}_{n_k-r_{1k}})^2$:Depending on the responding part of sample $S_{n_k}$, the sample mean square of auxiliary variable X for the kth stratum.

$s^{*^2}_{x_{m_k}}=\frac{1}{m_k-r_{2k}-1}\sum ^{m_k-r_{2k}}_{j=1}(x_{kj}-\bar{x}_{m_k-r_{2k}})^2$: Depending on the responding part of sample $S_{m_k}$, the sample mean square of auxiliary variable X for the kth stratum.

$s^{*^2}_{y_{n_k}}=\frac{1}{n_k-r_{1k}-1}\sum ^{n_k-r_{1k}}_{j=1}(y_{kj}-\bar{y}_{n_k-r_{1k}})^2$: Depending on the responding part of sample $S_{n_k}$, the sample mean square of study variable Y for the kth stratum.

$s^{*^2}_{y_{m_k}}=\frac{1}{m_k-r_{2k}-1}\sum ^{m_k-r_{2k}}_{j=1}(y_{kj}-\bar{y}_{m_k-r_{2k}})^2$: Depending on the responding part of sample $S_{m_k}$, the sample mean square of study variable Y for the kth stratum.

$s^{*^2}_{z_{n_k}}=\frac{1}{n_k-r_{1k}-1}\sum ^{n_k-r_{1k}}_{j=1}(z_{kj}-\bar{z}_{n_k-r_{1k}})^2$: Depending on the responding part of sample $S_{n_k}$, the sample mean square of auxiliary variable Z for the kth stratum.

$s^{*^2}_{z_{m_k}}=\frac{1}{m_k-r_{2k}-1}\sum ^{m_k-r_{2k}}_{j=1}(z_{kj}-\bar{z}_{m_k-r_{2k}})^2$: Depending on the responding part of sample $S_{m_k}$, the sample mean square of auxiliary variable Z for the kth stratum.

$W_k$ =$\frac{N_k}{N}$: The original weight of the kth stratum, k= 1, 2,....,L

$W_k^*$: The weight obtained by calibration of the kth stratum, k= 1, 2,....,L

$Q_k$: The independent weight of the kth stratum, k= 1, 2,....,L

## Non-response probability model

The *k*th stratum is considered based on the random non-response model proposed by Singh and Joarder^28^. In the first phase, a sample of size $n_k$ taken from the population, $n_k-r_{1k}$ units responded, while random non-response was observed on the remaining $r_{1k}$ units, where $r_{1k}$ may have any value from the set $\{0,1,2,...,(n_k-2)\}$. Again, in the second phase, from the $n_k-r_{1k}$ respondent units, $m_k-r_{2k}$ units responded, and $r_{2k}$ do not respond, where $r_{2k}$ falls within the range $\{0,1,2,..., (m_k-2)\}$. It is assumed that $r_{jk}\ge 0$, $j= 1, 2$ and $r_{1k}\le (n_k-2)$, $r_{2k}\le (m_k-2)$. Non-response may have possible values of $(n_k-2)$ and $(m_k-2)$ in the samples $S_{n_k}$ and $S_{m_k}$, respectively. These probabilities will be referred to as $p_1$ and $p_2$. The total number of ways to obtain $r_{jk}$ ($j=1, 2$) non-responses is $$\left( {\begin{array}{c}n_k-2\\ r_{1k}\end{array}}\right)$$ and $$\left( {\begin{array}{c}m_k-2\\ r_{2k}\end{array}}\right)$$. Then, the discrete random variables $r_{1k}$ and $r_{2k}$ have the corresponding probability distributions shown below:

$$P(r_{1k})$$= $$\frac{n_k-r_{1k}}{n_kq_1+2p_1}\left( {\begin{array}{c}n_k-2\\ r_{1k}\end{array}}\right) p^{r_{1k}}_1q^{n_k-r_{1k}-2}_1 \hspace{6pt} ; r_{1k} =0, 1, 2,..., n_k-2$$ and

$$P(r_{2k})$$= $$\frac{m_k-r_{2k}}{n_kq_2+2p_2}\left( {\begin{array}{c}m_k-2\\ r_{2k}\end{array}}\right) p^{r_{2k}}_2q^{m_k-r_{2k}-2}_2 \hspace{6pt} ; r_{2k} = 0, 1, 2,..., m_k-2$$

where $$q_1=1-p_1$$ and $$q_2=1-p_2$$.

## Suggested estimator

A wide class of estimators that may be used to estimate the population variance are proposed as follows, assuming the impact of random non-response on both the study variable Y and the first auxiliary variable X.

1 $$\begin{aligned} T=\sum _{k=1}^{L}W_k^{*2}T_k \end{aligned}$$

where

2 $$\begin{aligned} T_k=f(s^{*2}_{y_{m_k}},s^{*2}_{x_{m_k}},h(s^{*2}_{x_{n_k}},s^{2}_{z_{n_k}})), k=1,2,...,L \end{aligned}$$

In this case, $h(s^{*2}_{x_{n_k}},s^{*2}_{z_{n_k}})$ is a class of estimators of $S^2_X$ based on information on $s^{*2}_{x_{n_k}}$ and $s^{2}_{z_{n_k}}$ such that h($S^2_X$,$S^2_Z$)=$S^2_X$.

As we proceed, we will examine the composite class of estimators applicable to individual strata in two-phase sampling.

3 $$\begin{aligned} T_k=f(s^{*2}_{y_{m_k}},s^{*2}_{x_{m_k}},h(s^{*2}_{x_{n_k}},s^{2}_{z_{n_k}}))=g(s^{*2}_{y_{m_k}},s^{*2}_{x_{m_k}},s^{*2}_{x_{n_k}},s^{2}_{z_{n_k}}) \end{aligned}$$

such that g$$(S^2_{Y_k},S^2_{X_k},S^2_{X_k},S^2_{Z_k})$$=$$S^2_{Y_k}$$

We assume that $g(s^{*2}_{y_{m_k}},s^{*2}_{x_{m_k}},s^{*2}_{x_{n_k}},s^{2}_{z_{n_k}})$ meets the regularity conditions listed below:

- Regardless of the sample chosen, the function $g(s^{*2}_{y_{m_k}},s^{*2}_{x_{m_k}},s^{*2}_{x_{n_k}},s^{2}_{z_{n_k}})$ takes on values within a closed convex subspace of the four-dimensional real space $R^4$ that includes the point $(S^2_{Y_k},S^2_{X_k},S^2_{X_k},S^2_{Z_k})$.
- In $R^4$, the function $g(s^{*2}_{y_{m_k}},s^{*2}_{x_{m_k}},s^{*2}_{x_{n_k}},s^{2}_{z_{n_k}})$ is continuous and bounded.
- The partial derivatives of $g(s^{*2}_{y_{m_k}},s^{*2}_{x_{m_k}},s^{*2}_{x_{n_k}},s^{2}_{z_{n_k}})$ of the first, second, and third orders exist and are continuous and bounded in $R^4$.

The class of estimators $T_k$ is extensive, as any parametric function $g(s^{*2}_{y_{m_k}},s^{*2}_{x_{m_k}},s^{*2}_{x_{n_k}},s^{2}_{z_{n_k}})$ that meets the stated regularity conditions, and has g$(S^2_{Y_k},S^2_{X_k},S^2_{X_k},S^2_{Z_k})=S^2_{Y_k}$, may generate estimators for the population mean square of each stratum. Several examples of this class of estimators are:

$$T_{1k}=\frac{s^{*2}_{y_{m_k}}}{s^{*2}_{x_{m_k}}}\bigg [\frac{s^{*2}_{x_{n_k}}}{s^{*2}_{z_{n_k}}}\bigg ]s^{2}_{Z_{k}}$$,     $$T_{2k}= \frac{s^{*2}_{y_{m_k}}}{s^{*2}_{x_{m_k}}}\bigg [\frac{s^{*2}_{x_{n_k}}s^{*2}_{z_{n_k}}}{s^{2}_{Z_{k}}}\bigg ]$$,     $$T_{3k}=s^{*2}_{y_{m_k}}+b_1\big [\big (s^{*2}_{x_{n_k}}+b_2(s^{2}_{Z_{k}}-s^{*2}_{z_{n_k}}\big )-s^{*2}_{x_{m_k}}\big ]$$

$T_{4k}=\frac{s^{*2}_{y_{m_k}}}{s^{*2}_{y_{n_k}}}\big [s^{*2}_{x_{n_k}}+b_3(s^{2}_{Z_{k}}-s^{*2}_{z_{n_k}}\big ]$ where $b_1$, $b_2$ and $b_3$ are the true scalars.

$$T_{5k}=s^{*2}_{y_{m_k}}exp\biggl (\frac{s^{*2}_{x_{n_k}}-s^{*2}_{x_{m_k}}\frac{s^{2}_{Z_{k}}}{s^{*2}_{z_{n_k}}}}{s^{*2}_{x_{n_k}}+s^{*2}_{x_{m_k}}\frac{s^{2}_{Z_{k}}}{s^{*2}_{z_{n_k}}}}\biggl )$$     $$\forall k=1,2,...L$$

## Calibration techniques have been proposed to acquire the optimum strata weights

The new calibration estimator of the population variance under stratified sampling is provided by

$$\begin{aligned} T=\sum _{k=1}^{L}W_k^{*2}T_k \end{aligned}$$

where $T_k=f(s^{*2}_{y_{m_k}},s^{*2}_{x_{m_k}},h(s^{*2}_{x_{n_k}},s^{2}_{z_{n_k}})), k=1,2,...,L$ and we obtain the calibrated strata weights $W_k^*$, where k$\in \{1,2,...,L\}$.

Based on the following calibration requirements, the distance function (chi-square type) $\sum _{k=1}^{L}\frac{(W_k^*-W_k)^2}{Q_kW_k}$ is minimized:

1. $$\sum _{k=1}^{L}W_k^*$$=1
2. $$\sum _{k=1}^{L}W_k^*c_{z_k}$$=$$C_{Z}$$
3. $$\sum _{k=1}^{L}W_k^*c_{{x}_{m_k-r_{2k}}}$$= $$\sum _{k=1}^{L}W_kc_{{x}_{n_k-r_{1k}}}$$

where, $$c_{z_k}$$=$$\frac{s_{z_k}}{\bar{z_k}}$$, $$C_Z$$=$$\frac{S_Z}{\bar{Z}}$$, $$c_{{x}_{n_k-r_{1k}}}$$=$$\frac{s_{{x}_{n_k-r_{1k}}}}{\bar{x}_{n_k-r_{1k}}}$$ and $$c_{{x}_{m_k-r_{2k}}}$$=$$\frac{s_{{x}_{m_k-r_{2k}}}}{\bar{x}_{m_k-r_{2k}}}$$.

It is important to note that $Q_k > 0$ are appropriately determined weights that will determine the estimator form.

In Appendix A, detailed derivations have been given.

### Bias and mean square error of the suggested estimator

We utilize the transformations provided below while taking into account large sample assumptions to analyze the properties of estimator T:

$$\begin{aligned} s^{*2}_{y_{m_k}}=S^2_{Y_k}(1+\varepsilon _{0k}),\hspace{6pt} s^{*2}_{x_{m_k}}=S^2_{X_k}(1+\varepsilon _{1k}),\hspace{6pt} s^{*2}_{z_{n_k}}=S^2_{Z_k}(1+\varepsilon _{2k}),\hspace{6pt} s^{*2}_{x_{n_k}}=S^2_{X_k}(1+\varepsilon _{3k}) \end{aligned}$$

such that $|\epsilon _{ik}| \le$1, $\forall$i= 0, 1, 2, 3 and $E(\epsilon _{ik})$= 0.

According to calculations, the Bias(T) and the MSE(T) of the suggested estimator T, which are accurate to the first order of approximation, are as follows:

4 $$\begin{aligned} \begin{aligned} Bias(T)= \frac{1}{2}\sum _{k=1}^{L}W^{*2}_k\begin{bmatrix}S^4_{X_k}\big (f_{1k}d_{22k}+d_{33k}f_{3k}+2d_{23k}f_{3k}\big )C^2_{1k}+2S^2_{Y_k}S^2_{X_k}\rho _{01k}\big (d_{12k}f_{1k}+d_{13k}f_{3k}\big )\\ +2S^2_{X_k}S^2_{Z_k}\rho _{12k}\big (d_{24k}f_{2k}+d_{34k}f_{2k}\big ) +2S^2_{Y_k}S^2_{Z_k}d_{14k}f_{2k}\rho _{02k} +S^4_{Z_k}d_{44}f_{2k}C^2_{2k}\end{bmatrix} \end{aligned} \end{aligned}$$

and

5 $$\begin{aligned} MSE(T)=\sum _{k=1}^{L}W^{*4}_k\begin{bmatrix}S^4_{Y_k}f_{1k}C^2_{0k}+d^2_{2k}S^4_{X_k}C^2_{1k}f_{4k}+d^2_{4k}S^4_{Z_k}f_{2k}C^2_{2k} +2d_{4k}\rho _{02k}f_{2k}S^2_{Y_k}S^2_{Z_k}+2S^2_{Y_k}S^2_{X_k}d_{2k}\rho _{01k}f_{4k}\end{bmatrix} \end{aligned}$$

where

$$C^2_{0k}$$=$$\lambda _{400k}$$-1,                   $$C^2_{1k}$$=$$\lambda _{040k}$$-1,                 $$C^2_{2k}$$=$$\lambda _{004k}$$-1

$$\rho _{01k}=\lambda _{220k}$$-1,                 $$\rho _{02k}=\lambda _{202k}$$-1,               $$\rho _{12k}=\lambda _{022k}$$-1

$$f_{1k}$$=$$\bigg (\frac{1}{m_kq_2+2p_2}-\frac{1}{N_k}\bigg )$$,      $$f_{2k}$$=$$\bigg (\frac{1}{n_k}-\frac{1}{N_k}\bigg )$$,            $$f_{3k}$$=$$\bigg (\frac{1}{n_kq_1+2p_1}-\frac{1}{N_k}\bigg )$$

and

$$\begin{aligned} \lambda _{\alpha \beta \gamma k}=\frac{\mu _{\alpha \beta \gamma k}}{\sqrt{\mu ^{\alpha }_{200k}\mu ^{\beta }_{020k}\mu ^{\gamma }_{002k}}},~~~\mu _{\alpha \beta \gamma k}=\frac{1}{N_k}\sum _{j=1}^{N_k}(Y_{kj}-\bar{Y}_k)^{\alpha }(X_{kj}-\bar{X}_k)^{\beta }(Z_{kj}-\bar{Z}_k)^{\gamma } \end{aligned}$$

Appendix B has detailed derivations.

### The suggested estimator’s minimum mean square error under optimal condition.

We note from Eq. (5) that the derivatives $d_{2k}$ and $d_{4k}$ have an impact on the MSE of the estimator T. So, in order to acquire the derivatives’ optimal values, we minimize the MSE concerning them as follows:

6 $$\begin{aligned} d_{{2k}_{opt}}=-\frac{\rho _{01k}}{C^2_{1k}}\frac{S^2_{Y_k}}{S^2_{X_k}} \end{aligned}$$

and

7 $$\begin{aligned} d_{{4k}_{opt}}=-\frac{\rho _{02k}}{C^2_{2k}}\frac{S^2_{Y_k}}{S^2_{Z_k}} \end{aligned}$$

We may obtain the minimum mean square error (Min. MSE)) of the estimator T by substituting the optimal values of $d_{{2k}_{opt}}$ and $d_{{4k}_{opt}}$ from Eqs. (6) and (7), respectively, in Eq. (5) as follows:

8 $$\begin{aligned} Min. MSE(T)=\sum _{k=1}^{L}W_k^{*4}S^4_{Y_k}\Bigg [f_{1k}C^2_{0k}-\frac{\rho ^2_{01k}}{C^2_{1k}}f_{4k}-\frac{\rho ^2_{02k}}{C^2_{2k}}f_{2k}\Bigg ] \end{aligned}$$

### Effect of measurement error

Y and X actual and observed values are denoted by $y_{{kj}_a}$, $x_{{kj}_a}$, and $y_{{kj}_o}$, $x_{{kj}_o}$, while $u_{kj}$, and $v_{kj}$ denote the corresponding measurement errors. Then $x_{{kj}_a}= x_{{kj}_o}+v_{kj}$ and $y_{{kj}_a}= y_{{kj}_o}+u_{kj}$, resulting in $V(y_{{kj}_a})=V(y_{{kj}_o})+V(u_{kj})$, with zero covariance term because the errors are independent.

This implies $s^2_{y_{ka}}=s^2_{y_{ko}}+s^2_{u_{k}}$, so that $MSE(s^2_{y_{ka}})=MSE(s^2_{y_{ko}})+MSE(s^2_{u_{k}})$.

$$\begin{aligned} \therefore Min.MSE(T)&=\sum _{k=1}^{L}MSE(s^2_{y_{ka}})\\&=\sum _{k=1}^{L}\bigg [MSE(s^2_{y_{ko}})+M(s^2_{u_{k}})\bigg ]\\&=\sum _{k=1}^{L}MSE(s^2_{y_{ko}})+\sum _{k=1}^{L}MSE(s^2_{u_{k}}) \end{aligned}$$

The expression for Min.MSE was determined as follows: measurement errors occurred only on the study variable Y and the primary auxiliary variable X, not on the secondary auxiliary variable Z.

9 $$\begin{aligned} Min.MSE(T)=\sum _{k=1}^{L}W_k^{*4}S^4_{Y_k}\Bigg [f_{1k}C^2_{0k}-\frac{\rho ^2_{01k}}{C^2_{1k}}f_{4k}-\frac{\rho ^2_{02k}}{C^2_{2k}}f_{2k}\Bigg ]+\sum _{k=1}^{L}W_k^{*4}S^4_{u_k}f_{1k}C^{'2}_{0k} \end{aligned}$$

where

$$\begin{aligned} C^{'2}_{0k}=\lambda ^{'}_{40k}-1, ~~~ \lambda ^{'}_{40k}=\frac{\mu ^{'}_{40k}}{\sqrt{\mu _{20k}^{'2}}} ~~~and ~~~\mu _{abk}^{'}=\frac{1}{N_k}\sum _{j=1}^{N_k}(u_{kj}-\bar{u}_{k})^a(v_{kj}-\bar{v}_{k})^b \end{aligned}$$

## Numerical study

An estimator’s performance must first be evaluated in terms of its characteristics before it may be used in practical scenarios. Therefore, an empirical investigation has been conducted in this part using both real and simulated data for the suggested estimator.

**Table 1:** Population parameters..

Parameters	Real data				Simulated data			
	Stratum 1	Stratum 2	Stratum 3	Stratum 4	Stratum 1	Stratum 2	Stratum 3	Stratum 4
$N_k$	2500	2600	2311	8000	10000	15000	5000	8000
$n_k$	875	910	809	800	3000	3000	2000	800
$m_k$	625	650	578	500	2000	2000	1200	500
$n_{k-r_{1k}}$	750	780	693	750	2500	2500	1600	750
$m_{k-r_{2k}}$	500	520	462	450	1600	1600	900	450
$\rho _{xyk}$	0.90	0.89	0.97	0.6	0.9	0.8	0.8	0.6
$\rho _{xzk}$	0.83	0.92	0.93	0.6	0.9	0.8	0.8	0.6
$\rho _{yzk}$	0.75	0.83	0.92	0.6	0.9	0.8	0.8	0.6

We are comparing the suggested estimator T and the contemporary estimator $\tau$ to see how well they perform in random non-response. The estimator $\tau$ is defined as follows:

$$\tau$$=$$\sum ^{L}_{k=1}W^{*2}_ks^{*2}_{y_{m_k}}$$

Additionally, we are comparing these estimators with the standard estimator since it is the only available option when dealing with non-response and measurement errors.

The following are the expressions for its MSE, with and without measurement errors, respectively:

10 $$\begin{aligned} MSE(\tau )=\sum _{k=1}^{L}W_k^{*4}S^4_{Y_{N_k}}\bigg (\frac{1}{m_kq_2+2p_2}-\frac{1}{N_k}\bigg )(\lambda _{400k}-1) \end{aligned}$$

and

11 $$\begin{aligned} MSE(\tau )=\sum _{k=1}^{L}W_k^{*4}S^4_{Y_{N_k}}\bigg (\frac{1}{m_kq_2+2p_2}-\frac{1}{N_k}\bigg )(\lambda _{400k}-1)+\sum _{k=1}^{L}W_k^{*4}S^4_{uk}f_{1k}C^{'2}_{0k} \end{aligned}$$

The Percentage Relative Efficiency (PRE) of the proposed estimator T concerning the estimator $\tau$ is given by

PRE=$$\frac{MSE(\tau )}{Min.MSE(T)}*100$$

Where Eqs. (8)–(11) give the corresponding equations for Min MSE(T) and MSE($\tau$), without or with measurement errors, respectively.

The following $Q_k$ values have been taken into consideration:

Case A: $$Q_k$$=1.0

Case B: $$Q_k$$=$$\frac{1}{W_k}$$

Case C: $$Q_k$$=$$\bar{Z_k}$$

Case D: $$Q_k$$=$$S^2_{Z_{N_k}}$$

The calibrated stratum weights and PREs, resulting from both the presence and absence of non-response, are displayed in the tables below, for both simulated and real data.

### Study based on simulated data

We conducted a simulation relevant to our theoretical findings using the statistical computing software R. To achieve our objectives, we used the $MASS$ package’s function $mvrnorm$ to generate data from poisson distributions with given parameters and a given correlation coefficient for the study and the auxiliary variables. To generate data from other acceptable distributions, use the function $genCorGen$ included in the package $simstudy$. The measurement errors were generated using a univariate standard normal distribution with the function $rnorm$. Table 1 shows the population parameters for the generated data.

The resulting calibrated stratum weights and PREs in presence of non-response and in absence of non-response are shown in Tables 2, 3 and 4, respectively.

**Table 2:** Calibrated strata weights for simulated data..

Case	Stratum	$Q_k$	$W_k$	$W_k^*$
A	1	1	0.2631579	0.44807455
	2	1	0.3947368	0.36962453
	3	1	0.1315789	0.13017106
	4	1	0.2105263	0.05212985
B	1	3.800	0.2631579	0.43883647
	2	2.533	0.3947368	0.38908713
	3	7.600	0.1315789	0.13022077
	4	4.750	0.2105263	0.04185563
C	1	24.990	0.2631579	0.44804409
	2	25.009	0.3947368	0.36968872
	3	25.060	0.1315789	0.13017123
	4	24.970	0.2105263	0.05209596
D	1	25.370	0.2631579	0.44812328
	2	24.740	0.3947368	0.36952187
	3	24.860	0.1315789	0.13017080
	4	25.300	0.2105263	0.05218405

**Table 3:** PRE of T w.r.t. $\tau$ for simulated poisson data.

$p_1$	$p_2$	In the absence of measurement error				In the presence of measurement error			
		Case A	Case B	Case C	Case D	Case A	Case B	Case C	Case D
0.05	0.05	123.0381	122.6552	123.0369	123.0401	122.9925	122.6105	122.9913	122.9945
0.05	0.10	123.1304	122.7438	123.1291	123.1323	123.0845	122.6989	123.0833	123.0865
0.05	0.15	123.2208	122.8308	123.2195	123.2227	123.1747	122.7857	123.1735	123.1767
0.05	0.20	123.3094	122.9161	123.3081	123.3114	123.2631	122.8707	123.2619	123.2651
0.10	0.05	121.7219	121.3717	121.7208	121.7237	121.6794	121.3300	121.6783	121.6811
0.10	0.10	121.8941	121.5380	121.8929	121.8959	121.8511	121.4959	121.8500	121.8529
0.10	0.15	122.0630	121.7013	122.0619	122.0649	122.0197	121.6588	122.0185	122.0215
0.10	0.20	122.2289	121.8617	122.2277	122.2308	122.1852	121.8189	122.1840	122.1870
0.15	0.05	120.2839	119.9687	120.2828	120.2855	120.2446	119.9302	120.2436	120.2462
0.15	0.10	120.5415	120.2182	120.5404	120.5431	120.5016	120.1791	120.5006	120.5033
0.15	0.15	120.7947	120.4634	120.7936	120.7963	120.7542	120.4238	120.7532	120.7559
0.15	0.20	121.0436	120.7046	121.0425	121.0453	121.0026	120.6644	121.0015	121.0043
0.20	0.05	118.7063	118.4288	118.7054	118.7077	118.6706	118.3937	118.6697	118.6720
0.20	0.10	119.0554	118.7673	119.0544	119.0568	119.0189	118.7314	119.0179	119.0203
0.20	0.15	119.3990	119.1007	119.3981	119.4005	119.3618	119.0641	119.3608	119.3633
0.20	0.20	119.7374	119.4290	119.7364	119.7389	119.6993	119.3917	119.6984	119.7009

**Table 4:** In the absence of non-response, PRE is observed from simulated data when $p_1 = p_2 = 0$..

Stratum	PRE (In the absence of measurement error)	PRE (In the presence of measurement error)
Case I	124.2297	124.1813
Case II	123.8176	123.7701
Case III	124.2284	124.18
Case IV	124.2318	124.1834

### Study based on real data

The information in this section demonstrates the practical application of the proposed class of estimators. The dataset utilized is accessible within the UCI machine learning repository, titled “Gas Turbine CO and NOx Emission Data Set.” This dataset comprises 36,733 instances featuring 11 sensor measurements from a gas turbine situated in the northwestern region of Turkey, aggregated over an hour using average or sum calculation methods for the analysis of CO and NOx (NO + NO2) flue gas emissions. To conduct the analysis mentioned above, the specific file utilized is $gt_{2011}.csv$.

We employed the subsequent set of primary and auxiliary variables in this study:

Y: Gas turbine exhaust pressure (GTEP)

X: Air filter difference pressure (AFDP)

Z: Turbine inlet temperature (TIT)

The stratification is organized based on the Ambient temperature (AT) in the following manner:

Stratum 1: from 2.1163-12.707 C

Stratum 2: from 12.708-21.759 C

Stratum 3: from 21.760-34.532 C

In real-world circumstances, the goal is to estimate the variance as precisely as possible. However, complete data is typically not always available. Therefore, we consider the case where some data on the study variable is unavailable. The statistical characteristics of the population are detailed in Table 1, while the calibrated weights for the strata are listed in Table 5. The PRE (Precentage Relative Efficiency) for both the non-response and absence of non-response cases is presented in Tables 6 and 7, respectively.

**Table 5:** Calibrated strata weights for real data.

Case	Stratum	$Q_k$	$W_k$	$W_k^*$
A	1	1	0.2061856	0.3209746
	2	1	0.6185567	0.5243500
	3	1	0.1752577	0.1546754
B	1	4.85	0.2061856	0.2566935
	2	1.6167	0.6185567	0.5356455
	3	5.7059	0.1752577	0.2076610
C	1	0.00092	0.2061856	0.3258734
	2	0.00092	0.6185567	0.5187164
	3	0.0009196857	0.1752577	0.1554102
D	1	0.0045425256	0.2061856	0.1816363
	2	0.0031285169	0.6185567	0.6199598
	3	0.0046467310	0.1752577	0.1984039

**Table 6:** PRE of T w.r.t. $\tau$ for real data.

$p_1$	$p_2$	In the absence of measurement error				In the presence of measurement error			
		Case A	Case B	Case C	Case D	Case A	Case B	Case C	Case D
0.05	0.05	264.5064	280.9632	261.8853	293.3473	262.4120	278.5422	259.8380	290.6966
0.05	0.10	268.6831	284.3495	266.1682	296.1447	266.5021	281.8539	264.0328	293.430
0.05	0.15	272.8552	287.7074	270.4512	298.9049	270.5860	285.1367	268.2260	296.1271
0.05	0.20	277.0228	291.0372	274.7343	301.6286	274.6639	288.3911	272.4175	298.7875
0.10	0.05	241.0445	254.7055	238.8611	264.8419	239.4063	252.8268	237.2576	262.7989
0.10	0.10	246.0068	259.1428	243.8921	268.9041	244.2764	257.1770	242.1959	266.7791
0.10	0.15	251.0106	263.5895	248.9701	272.9584	249.1849	261.5345	247.1779	270.7499
0.10	0.20	256.0565	268.0456	254.0956	277.0046	254.1324	265.8995	252.2041	274.7114
0.15	0.05	219.3091	230.6233	217.4957	238.9029	218.0470	229.1854	216.2590	237.3481
0.15	0.10	224.8071	235.7881	223.0355	243.8426	223.4541	234.2603	221.7079	242.1996
0.15	0.15	230.4004	241.0149	228.6758	248.8245	228.9519	239.3935	227.2525	247.0903
0.15	0.20	236.0913	246.3046	234.4191	253.8493	234.5427	244.5859	232.8954	252.0208
0.20	0.05	199.1163	208.4569	197.6162	215.1987	198.1634	207.3767	196.6817	214.0359
0.20	0.10	204.9445	214.0888	203.4670	220.7086	203.9063	212.9220	202.4475	219.4593
0.20	0.15	210.9229	219.8402	209.4726	226.3199	209.7939	218.5820	208.3624	224.9796
0.20	0.20	217.0573	225.7149	215.6391	232.0353	215.8316	224.3602	214.4322	230.5994

**Table 7:** In the absence of non-response, PRE is observed from real data when $p_1$ = $p_2$ = 0.

Stratum	PRE (In the absence of measurement error)	PRE (In the presence of measurement error)
Case I	286.7866	284.2111
Case II	307.7331	304.693
Case III	283.4925	280.983
Case IV	323.7506	320.3699

## Discussion

After conducting a detailed numerical study, we have identified the following key points:

1. The strata weights produced by the calibration procedures exhibit slight discrepancies from the actual ones, as evident in Tables 2 and 5. Nevertheless, our findings indicate that the calibration technique effectively enhances the stratum weights, resulting in more accurate estimates.
2. Table 3 reveals a consistent pattern: when $p_1$, $p_2$ $\in$(0.05, 0.1), the suggested estimator consistently outperforms the existing estimator, regardless of the presence or absence of measurement errors. This observation is further supported by the real data presented in Table 6.
3. Further analysis of Tables 3 and 6 reveals that an increase in the value of $p_2$, while keeping $p_1$ constant, results in a higher PRE. This observation is a significant outcome of our research. Additionally, when $p_2$ remains fixed and $p_1$ increases, the PRE decreases, aligning with our expectations.
4. Tables 4 and 7 demonstrate that the proposed estimator yields a higher Percentage Relative Efficiency (PRE) than the conventional estimator in the absence of non-response also, underscoring the effectiveness of our method, even without non-response.
5. It is noteworthy that as the correlation coefficient’s value increases, the PRE also increases. Conversely, a decrease in the correlation coefficient leads to a decrease in PRE.

The recommended estimator successfully mitigates the adverse effects of random non-response and measurement errors in two-phase stratified sampling. When additional information on two positively related variables is available, the advantages are evident. We anticipate the evolution of more estimators within the proposed class, allowing survey statisticians to provide even more precise estimates.

## Conclusions

Our research has illuminated several critical contributions and practical applications:

The calibration technique significantly enhances the accuracy of stratum weights, leading to more precise estimates, even in the presence of minor deviations from the actual weights. The proposed estimator consistently outperforms its counterparts within specific parameter ranges, showcasing its robustness in handling measurement errors. The superior Percentage Relative Efficiency (PRE) of our proposed estimator, even in scenarios without non-response, highlights its effectiveness in improving estimation accuracy. We’ve observed that the correlation coefficient and the values of $p_1$ and $p_2$ play significant roles in the performance of the estimator. The versatility of our estimation approach extends its applicability across diverse fields, including the estimation of variance in simulated data. The results obtained from simulated data are further validated through the analysis of real-world data, such as gas turbine exhaust pressure, confirming the applicability and reliability of our proposed methodology in practical scenarios.

Our study provides valuable methodologies to enhance population variance estimation, particularly in practical scenarios rife with non-response and measurement errors. The consistent and outstanding performance of our proposed estimators corroborates their effectiveness and reliability within the domain of survey statistics. Moreover, incorporating neutrosophic statistics aligns with the need to address uncertainty and imprecision in survey data, further reinforcing the effectiveness of our proposed methodology. The validation of our simulated data against real-world datasets substantiates the applicability and trustworthiness of our proposed methodology in practical, real-life scenarios.

### Supplementary Information

Supplementary Information.

## Data Availability

Secondary data used in the manuscript is freely available from the UCI Machine Learning Repository dataset named ‘Gas Turbine CO and NOx Emission Data Set.’ For the above analysis, we have chosen the file $`gt_{2011}.csv'$. The data can be accessed from https://archive.ics.uci.edu/dataset/551/gas+turbine+co+and+nox+emission+data+set.
