# Supplementary material for: A general class of improved population variance estimators under non-sampling errors using calibrated weights in stratified sampling
Source: Sci Rep. 2024 Feb 5;14:2948. doi: 10.1038/s41598-023-47234-1 (PMC10844305; doi:10.1038/s41598-023-47234-1)
Supplement: Supplementary file 1 — Supplementary Information. [file 41598_2023_47234_MOESM1_ESM.pdf]

Supplementary material - A General Class of Improved Population Variance Estimators Under Non-Sampling Errors Using Calibrated Weights in Stratified Sampling

M. K. Pandey[1,*], G. N. Singh[1], Tolga Zaman[2], Aned Al Mutairi[3], and Manahil SidAhmed Mustafa[4]

[1]Department of Mathematics and Computing, Indian Institute of Technology (Indian School of Mines), Dhanbad-826004 (India)
[2]Gumushane University, faculty of health sciences, Turkey
[3]Department of Mathematical Sciences, College of Science, Princess Nourah Bint Abdulrahman University, P.O. Box 84428, Riyadh 11671, Saudi Arabia
[4]Department of Statistics, Faculty of Science, University of Tabuk, Tabuk, Saudi Arabia
[*]Corresponding author e-mail: maheshbabu3797@gmail.com

ABSTRACT

This paper proposes a new calibration estimator for population variance within a stratified two-phase sampling design. It takes into account random non-response and measurement errors, specifically applying this method to estimate the variance in Gas turbine exhaust pressure data. The study integrates additional information from two highly positively correlated auxiliary variables to develop a general class of estimators tailored for the stratified two-phase sampling scheme. The properties of these estimators, in terms of their biases and mean square errors, have been thoroughly examined and extensively analyzed through numerical and simulation studies. Furthermore, the calibrated weights of the strata are derived. The proposed estimators outperform the natural estimator of population variance. Finally, suitable recommendations have been made for survey statisticians intending to apply these findings to real-life problems.

Appendix A: Deriving calibrated strata weights

The task of determining the optimum calibrated weights W_k^* may be expressed as a nonlinear programming problem (NLPP) as shown below:

Minimize Z(W_1^*,W_2^*,...,W_L^*)=$\sum_{k=1}^{L} \frac{(W_k^*-W_k)^2}{Q_k W_k}$
subject to

$\sum_{k=1}^{L} W_k^*$=1
$\sum_{k=1}^{L} W_k^* c_{z_k}$=$C_Z$
$\sum_{k=1}^{L} W_k^* c_{x_{m_k-r_{2k}}} = \sum_{k=1}^{L} W_k c_{x_{n_k-r_{1k}}}$
and

$$W_k^* \geq 0; \quad k = 1,2,...,L. \tag{12}$$

Since the constraints are equality constraints, we can use the lagrange multipliers technique to solve the NLPP 12 and find the best solutions for W_k^* while ignoring the $W_k^* \geq 0$ restriction. The NLPP in 12 can be solved entirely if the values W_k^* satisfy the ignored restrictions.
Defining λ_1, λ_2, and λ_3 as lagrange multiplier, the lagrange function is given as:

$$L = \left[\sum_{k=1}^{L} \frac{(W_k^*-W_k)^2}{Q_k W_k} - 2\lambda_1\left(\sum_{k=1}^{L} W_k^* - 1\right) - 2\lambda_2\left(\sum_{k=1}^{L} W_k^* c_{z_k} - C_Z\right) - 2\lambda_3\left(\sum_{k=1}^{L} W_k^* c_{x_{m_k-r_{2k}}} - \sum_{k=1}^{L} W_k c_{x_{n_k-r_{1k}}}\right)\right] \tag{13}$$

The necessary and sufficient requirements for solving W_k^* are as follows in order to determine its optimal value:

$$\frac{\delta L}{\delta W_k^*} = 2\frac{W_k^* - W_k}{W_k Q_k} - 2\lambda_1 - 2\lambda_2 c_{z_k} - 2\lambda_3 c_{x_{m_k - r_{2k}}} \tag{14}$$

$$\frac{\delta L}{\delta \lambda_1} = -2\left(\sum_{k=1}^{L} W_k^* - 1\right) \tag{15}$$

$$\frac{\delta L}{\delta \lambda_2} = -2\left(\sum_{k=1}^{L} W_k^* c_{z_k} - C_Z\right) \tag{16}$$

$$\frac{\delta L}{\delta \lambda_3} = -2\left(\sum_{k=1}^{L} W_k^* c_{x_{m_k - r_{2k}}} - \sum_{k=1}^{L} W_k c_{x_{n_k - r_{1k}}}\right) = 0 \tag{17}$$

From equation 14 we have

$$W_k^* = W_k + (\lambda_1 + \lambda_2 c_{z_k} + \lambda_3 c_{x_{m_k - r_{2k}}}) W_k Q_k \tag{18}$$

If we put the value of W_k^* in equations 15, 16 & 17, we get...

$$\lambda_1 \sum_{k=1}^{L} W_k Q_k + \lambda_2 \sum_{k=1}^{L} W_k Q_k c_{z_k} + \lambda_3 \sum_{k=1}^{L} W_k Q_k c_{x_{m_k - r_{2k}}} = 1 - \sum_{k=1}^{L} W_k \tag{19}$$

$$\lambda_1 \sum_{k=1}^{L} W_k Q_k c_{z_k} + \lambda_2 \sum_{k=1}^{L} W_k Q_k c_{z_k}^2 + \lambda_3 \sum_{k=1}^{L} W_k Q_k c_{x_{m_k - r_{2k}}} c_{z_k} = C_Z - \sum_{k=1}^{L} W_k c_{z_k} \tag{20}$$

$$\lambda_1 \sum_{k=1}^{L} W_k Q_k c_{x_{m_k - r_{2k}}} + \lambda_2 \sum_{k=1}^{L} W_k Q_k c_{x_{m_k - r_{2k}}} c_{z_k} + \lambda_3 \sum_{k=1}^{L} W_k Q_k c_{x_{m_k - r_{2k}}}^2 = \sum_{k=1}^{L} W_k c_{x_{n_k - r_{1k}}} - \sum_{k=1}^{L} W_k c_{x_{m_k - r_{2k}}} \tag{21}$$

Let's write equations 19, 20, and 21 in matrix form

$$\begin{bmatrix} cal_a & cal_b & cal_c \\ cal_b & cal_e & cal_f \\ cal_c & cal_f & cal_h \end{bmatrix} \begin{bmatrix} \lambda_1 \\ \lambda_2 \\ \lambda_3 \end{bmatrix} = \begin{bmatrix} cal_d \\ cal_g \\ cal_i \end{bmatrix}$$

The solution of the above matrix provides the values of the lagrange multipliers, as stated below:

$$\lambda_1 = \frac{det_\alpha}{det}, \lambda_2 = \frac{det_\beta}{det}, \ \& \ \lambda_3 = \frac{det_\gamma}{det} \tag{22}$$

where

$$det = cal_a cal_e cal_h - cal_a cal_f^2 - cal_b^2 cal_h + 2 cal_b cal_c cal_f - cal_e cal_c^2 \tag{23}$$

$$det_\alpha = cal_d cal_e cal_h - cal_d cal_f^2 - cal_b cal_g cal_h + cal_b cal_i cal_f + cal_c cal_g cal_f - cal_c cal_i cal_e \tag{24}$$

$$det_\beta = cal_a cal_g cal_h - cal_a cal_i cal_f - cal_b cal_d cal_h + cal_c cal_d cal_f + cal_b cal_c cal_i - cal_c^2 cal_g \tag{25}$$

$$det_{\gamma} = cal_a cal_e cal_i - cal_a cal_g cal_f - cal_b^2 cal_i + cal_b cal_c cal_g + cal_b cal_d cal_f - cal_c cal_d cal_e \tag{26}$$

Now, let us define the term cal_a, cal_b, cal_c, cal_d, cal_e, cal_f, cal_g, cal_h, cal_i as follows:

$cal_a=\sum_{k=1}^{L} W_k Q_k$	$cal_b=\sum_{k=1}^{L} W_k Q_k c_{z_k}$
$cal_c=\sum_{k=1}^{L} W_k Q_k c_{x_{m_k-r_{2k}}}$	$cal_d=1-\sum_{k=1}^{L} W_k$
$cal_e=\sum_{k=1}^{L} W_k Q_k c_{z_k}^2$	$cal_f=\sum_{k=1}^{L} W_k Q_k c_{x_{m_k-r_{2k}}} c_{z_k}$
$cal_g=C_Z - \sum_{k=1}^{L} W_k c_{z_k}$	$cal_h=\sum_{k=1}^{L} W_k Q_k c_{x_{m_k-r_{2k}}}^2$
$cal_i=\sum_{k=1}^{L} W_k c_{x_{n_k-r_{1k}}} - \sum_{k=1}^{L} W_k c_{x_{m_k-r_{2k}}}$	

Appendix B: Deriving bias and MSE of the proposed estimator

The expectations obtained after applying the transformations specified in section 5.1 are as follows:

$E(\varepsilon_{0k}^2)=f_{1k}C_{0k}^2$	$E(\varepsilon_{1k}^2)=f_{1k}C_{1k}^2$	$E(\varepsilon_{2k}^2)=f_{2k}C_{2k}^2$
$E(\varepsilon_{3k}^2)=f_{3k}C_{1k}^2$	$E(\varepsilon_{0k}\varepsilon_{1k})=f_{1k}\rho_{01k}$	$E(\varepsilon_{0k}\varepsilon_{2k})=f_{2k}\rho_{02k}$
$E(\varepsilon_{0k}\varepsilon_{3k})=f_{3k}\rho_{01k}$	$E(\varepsilon_{1k}\varepsilon_{2k})=f_{2k}\rho_{12k}$	$E(\varepsilon_{1k}\varepsilon_{3k})=f_{3k}C_{1k}^2$
$E(\varepsilon_{2k}\varepsilon_{3k})=f_{2k}\rho_{12k}$		

Where the notations were previously defined in section 5.1.

$C_{0k}^2 = \lambda_{400k} - 1$	$C_{1k}^2 = \lambda_{040k} - 1$	$C_{2k}^2 = \lambda_{004k} - 1$
$\rho_{01k} = \lambda_{220k} - 1$	$\rho_{02k} = \lambda_{202k} - 1$	$\rho_{12k} = \lambda_{022k} - 1$
$f_{1k} = \left(\frac{1}{m_k q_2 + 2p_2} - \frac{1}{N_k}\right)$	$f_{2k} = \left(\frac{1}{n_k} - \frac{1}{N_k}\right)$	$f_{3k} = \left(\frac{1}{n_k q_1 + 2p_1} - \frac{1}{N_k}\right)$
$f_{4k} = f_{1k} - f_{3k}$		

$$\text{and} \quad \lambda_{\alpha\beta\gamma k} = \frac{\mu_{\alpha\beta\gamma k}}{\sqrt{\mu_{200k}^{\alpha}\mu_{020k}^{\beta}\mu_{002k}^{\gamma}}}, \quad \mu_{\alpha\beta\gamma k} = \frac{1}{N_k}\sum_{j=1}^{N_k}(Y_{kj}-\bar{Y}_k)^{\alpha}(X_{kj}-\bar{X}_k)^{\beta}(Z_{kj}-\bar{Z}_k)^{\gamma}$$

Remark. When the data are free of random nonresponse, or for p_1=0 and p_2=0, the above assumptions agree with the typical results.

The class of estimators T is now expressed in terms of ε's by expanding $g(s_{y_{m_k}}^{*2}, s_{x_{m_k}}^{*2}, s_{x_{n_k}}^{*2}, s_{z_{n_k}}^{2})$ about the point $g(S_{Y_k}^2, S_{X_k}^2, S_{X_k}^2, S_{Z_k}^2)$ using Taylor series expansion of second order. We get:

$$\begin{aligned} T_h = {} & g(S_{Y_k}^2, S_{X_k}^2, S_{X_k}^2, S_{Z_k}^2) + d_{1k}(s_{y_{m_k}}^{*2} - S_{Y_k}^2) + d_{2k}(s_{x_{m_k}}^{*2} - S_{X_k}^2) + d_{3k}(s_{x_{n_k}}^{*2} - S_{X_k}^2) + d_{1k}(s_{z_{n_k}}^{*2} - S_{Z_k}^2) \\ & + \frac{1}{2}\left[\begin{array}{c} d_{11k}(s_{y_{m_k}}^{*2} - S_{Y_k}^2 + d_{22k}(s_{x_{m_k}}^{*2} - S_{X_k}^2) + d_{33k}(s_{x_{n_k}}^{*2} - S_{X_k}^2) + d_{44k}(s_{z_{n_k}}^{*2} - S_{Z_k}^2) + 2d_{12k}(s_{y_{m_k}}^{*2} - S_{Y_k}^2)(s_{x_{m_k}}^{*2} - S_{X_k}^2) \\ +2d_{13k}(s_{y_{m_k}}^{*2} - S_{Y_k}^2)(s_{x_{n_k}}^{*2} - S_{X_k}^2) + 2d_{14k}(s_{y_{m_k}}^{*2} - S_{Y_k}^2)(s_{z_{n_k}}^{*2} - S_{Z_k}^2) \\ +2d_{23k}(s_{x_{m_k}}^{*2} - S_{X_k}^2)(s_{x_{n_k}}^{*2} - S_{X_k}^2) + 2d_{24k}(s_{x_{m_k}}^{*2} - S_{X_k}^2)(s_{z_{n_k}}^{*2} - S_{Z_k}^2) + 2d_{12k}(s_{x_{n_k}}^{*2} - S_{X_k}^2)(s_{z_{n_k}}^{*2} - S_{Z_k}^2) \end{array}\right] \\ & + \cdots \end{aligned} \tag{27}$$

where

$$d_{1k} = \left.\frac{\delta g(s_{y_{m_k}}^{*2}, s_{x_{m_k}}^{*2}, s_{x_{n_k}}^{*2}, s_{z_{n_k}}^{2})}{\delta s_{y_{m_k}}^{*2}}\right|_{g(S_{Y_k}^2, S_{X_k}^2, S_{X_k}^2, S_{Z_k}^2)}$$

$$d_{2k} = \left.\frac{\delta g(s_{y_{m_k}}^{*2}, s_{x_{m_k}}^{*2}, s_{x_{n_k}}^{*2}, s_{z_{n_k}}^{2})}{\delta s_{x_{m_k}}^{*2}}\right|_{g(S_{Y_k}^2, S_{X_k}^2, S_{X_k}^2, S_{Z_k}^2)}$$

$$d_{3k} = \left.\frac{\delta g(s_{y_{m_k}}^{*2}, s_{x_{m_k}}^{*2}, s_{x_{n_k}}^{*2}, s_{z_{n_k}}^{2})}{\delta s_{x_{n_k}}^{*2}}\right|_{g(S_{Y_k}^2, S_{X_k}^2, S_{X_k}^2, S_{Z_k}^2)}$$

$$d_{4k} = \left.\frac{\delta g(s^{*2}_{y_{m_k}}, s^{*2}_{x_{m_k}}, s^{*2}_{x_{n_k}}, s^2_{z_{n_k}})}{\delta s^{*2}_{z_{n_k}}}\right|_{g(S^2_{Y_k}, S^2_{X_k}, S^2_{X_k}, S^2_{Z_k})}$$

and d_{11k}, d_{22k}, d_{33k}, d_{44k}, d_{12k}, d_{13k}, d_{14k}, d_{23k}, d_{24k}, d_{34k} are the second order partial derivatives of $g(s^{*2}_{y_{m_k}}, s^{*2}_{x_{m_k}}, s^{*2}_{x_{n_k}}, s^2_{z_{n_k}})$ about g$(S^2_{Y_k}, S^2_{X_k}, S^2_{X_k}, S^2_{Z_k})$.

The condition g$(S^2_{Y_k}, S^2_{X_k}, S^2_{X_k}, S^2_{Z_k})$=$S^2_{Y_k}$ implies that

$$\left.\frac{\delta g(s^{*2}_{y_{m_k}}, s^{*2}_{x_{m_k}}, s^{*2}_{x_{n_k}}, s^2_{z_{n_k}})}{\delta s^{*2}_{y_{m_k}}}\right|_{g(S^2_{Y_k}, S^2_{X_k}, S^2_{X_k}, S^2_{Z_k})} = 1$$

that is

$$d_{1k} = 1$$

and

$$\left.\frac{\delta^2 g(s^{*2}_{y_{m_k}}, s^{*2}_{x_{m_k}}, s^{*2}_{x_{n_k}}, s^2_{z_{n_k}})}{\delta (s^{*2}_{y_{m_k}})^2}\right|_{g(S^2_{Y_k}, S^2_{X_k}, S^2_{X_k}, S^2_{Z_k})} = 0$$

that is

$$d_{11k} = 0$$

Furthermore, we put an extra constraint

$$d_{3k} = -d_{2k}$$

Because it is unknown what the population mean square of each stratum, $S^2_{X_k}$ $(k = 1, 2, ...L)$, is. Consequently, expanding in terms of ε's and applying the conditions as mentioned above, Eq. 27 becomes

$$\begin{aligned} T_h = {} & S^2_{Y_k} + S^2_{Y_k}\varepsilon_{0k} + d_{2k}S^2_{X_k}(\varepsilon_{1k} - \varepsilon_{3k}) + d_{4k}S^2_{Z_k}\varepsilon_{2k} \\ & + \frac{1}{2}\begin{bmatrix} d_{22h}S^4_{X_k}\varepsilon^2_{1k} + d_{33k}S^4_{X_k}\varepsilon^2_{3k} + d_{44k}S^4_{Z_k}\varepsilon^2_{2k} + 2d_{12k}S^2_{Y_k}S^2_{X_k}\varepsilon_{0k}\varepsilon_{1k} + 2d_{13k}S^2_{Y_k}S^2_{X_k}\varepsilon_{0k}\varepsilon_{3k} \\ +2d_{14k}S^2_{Y_k}S^2_{Z_k}\varepsilon_{0k}\varepsilon_{2k} + 2d_{23k}S^4_{X_k}\varepsilon_{1k}\varepsilon_{3k} + 2d_{24k}S^2_{X_k}S^2_{Z_k}\varepsilon_{1k}\varepsilon_{2k} + 2d_{12k}S^2_{X_k}S^2_{Z_k}\varepsilon_{2k}\varepsilon_{3k} \end{bmatrix} \end{aligned} \tag{28}$$

Using Eq. 28 in Eq. (1), we get:

$$T = \sum_{k=1}^{L} W^{*2}_k \begin{bmatrix} S^2_{Y_k} + S^2_{Y_k}\varepsilon_{0k} + d_{2k}S^2_{X_k}(\varepsilon_{1k} - \varepsilon_{3k}) + d_{4k}S^2_{Z_k}\varepsilon_{2k} \\ +\frac{1}{2}\begin{Bmatrix} d_{22k}S^4_{X_k}\varepsilon^2_{1k} + d_{33k}S^4_{X_k}\varepsilon^2_{3k} + d_{44k}S^4_{Z_k}\varepsilon^2_{2k} + 2d_{12k}S^2_{Y_k}S^2_{X_k}\varepsilon_{0k}\varepsilon_{1k} + 2d_{13k}S^2_{Y_k}S^2_{X_k}\varepsilon_{0k}\varepsilon_{3k} \\ +2d_{14k}S^2_{Y_k}S^2_{Z_k}\varepsilon_{0k}\varepsilon_{2k} + 2d_{23k}S^4_{X_k}\varepsilon_{1k}\varepsilon_{3k} + 2d_{24k}S^2_{X_k}S^2_{Z_k}\varepsilon_{1k}\varepsilon_{2k} + 2d_{12k}S^2_{X_k}S^2_{Z_k}\varepsilon_{2k}\varepsilon_{3k} \end{Bmatrix} \end{bmatrix} \tag{29}$$

The resultant equations for the Bias(T) and the MSE(T) of the proposed estimator T are obtained by assuming expectations on both sides of Eq. 29.
